# The C1QTNF6–MX2 Antiviral Axis Inhibits Porcine Circovirus Type 2 Replication in Porcine Macrophages

**DOI:** 10.3390/vetsci13010011

**Published:** 2025-12-21

**Authors:** Xiaolei Chen, Jiayao Jiang, Xiaocheng Bao, Chao Xu, Shuai Chen, Zhengchang Wu, Haifei Wang, Hairui Fan, Wenbin Bao

**Affiliations:** 1 College of Animal Science and Technology, Yangzhou University, Yangzhou 225009, China; mx120230917@stu.yzu.edu.cn (X.C.); mx120200807@stu.yzu.edu.cn (C.X.); zcwu@yzu.edu.cn (Z.W.); hyfiwang@yzu.edu.cn (H.W.); 2 Institute of Comparative Medicine, College of Veterinary Medicine, Yangzhou University, Yangzhou 225009, China; dx120220184@stu.yzu.edu.cn (J.J.); mx120240996@stu.yzu.edu.cn (X.B.); chenshuai@yzu.edu.cn (S.C.)

**Keywords:** C1QTNF6, immune responses, PCV2, ISGs, MX2

## Abstract

Porcine circovirus type 2 (PCV2), a widely distributed immunosuppressive virus, causes substantial economic losses in the global swine industry. *C1QTNF6* has emerged as a novel immunoregulatory factor attracting increasing research interest due to its dual roles in both pro-inflammatory and antiviral immune responses. However, the role of *C1QTNF6* in regulating PCV2 replication remains poorly characterized. Here, we analyzed *C1QTNF6* expression patterns and identified its highly specific expression in placental tissues in both humans and pigs. We then overexpressed *C1QTNF6* and conducted RNA sequencing analysis. Remarkably, 68 upregulated genes were identified, and most of them were interferon-stimulated genes (ISGs), including *MX2* and *ISG15*. Functional enrichment analysis revealed that these genes were primarily associated with defense response to viruses and antiviral innate immune response. Subsequently, experimental data show that PCV2 infection significantly suppressed inflammatory responses and markedly downregulated the expression of *C1qtnf6*, *MX2*, and *IFIT2*. Moreover, experimental data indicated that *C1QTNF6* inhibits PCV2 replication by targeting ISGs, while restoring *MX2* expression. To verify whether *C1QTNF6*-*MX2* antiviral axis mediates this antiviral effect, we constructed an *MX2* overexpression plasmid and demonstrated that *MX2* overexpression indeed significantly suppressed PCV2 replication. Together, these results provide important insights into PCV2-host interactions and the development of novel antiviral strategies.

## Simple Summary

Porcine circovirus type 2 (PCV2), the primary causative agent of postweaning multisystemic wasting syndrome, inflicts substantial economic losses on the global swine industry. *C1QTNF6* has emerged as a novel immunoregulatory factor attracting significant interest due to its dual functionality in mediating both pro-inflammatory and antiviral immune responses. In the study, we focused on the *C1QTNF6* on PCV2 infection in porcine alveolar macrophages (3D4/21). We found that *C1QTNF6* could inhibit PCV2 replication by activating ISGs. Our study provides important insights into PCV2-host interactions and the development of novel antiviral strategies.

## 1. Introduction

Porcine Circovirus Type 2 (PCV2) is an important pathogen causing widespread infection in pig populations. It has inflicted substantial economic losses on the global swine industry, thereby conferring significant value for immunological and virological research [1]. PCV2 belongs to the Circoviridae family and is a non-enveloped, single-stranded circular DNA virus [2]. Although the PCV2 genome is relatively small, its pathogenicity is complex, leading to a variety of clinical symptoms across different pig populations [3]. In recent years, the continuous advancement of molecular biology techniques has significantly advanced virological research, enabling researchers to obtain a deeper understanding of the pathogenic mechanisms of PCV2 and facilitating notable breakthroughs in the field of antiviral research. However, the mechanisms underlying PCV2 infection in host cells and the host’s antiviral immune responses remain poorly understood.

C1q and tumor necrosis factor-related protein 6 (*C1qtnf6*) are new highly homologous family members of adiponectin byproducts, involving four distinct domains: N-terminal signal peptide, short variable region, collagen-like domain, and C-terminal globular domain [4]. The collagen-like domain and N-terminal signal peptide domain are two structural domains that are commonly found in proteins involved in immune regulation, inflammation, and cellular signaling [5]. The exact role of *C1QTNF6* in antiviral activity has not yet been fully elucidated. Increasing evidence has revealed the complex role of *C1QTNF6* in immune responses, particularly its dual function in pro-inflammatory and antiviral immunity [6,7], positioning it as an emerging research hotspot. Studies have indicated that CTRP6 is associated with various pathological processes, including lipid metabolism [8], insulin resistance [9], tumors [10], inflammation [11], arthritis [12], and mitochondrial dysfunction [13]. As a novel immune regulatory factor, *C1QTNF6* is closely linked to classical immune mediators such as TNF-α [14] and IL-6 [15]. During ischemic stroke, the lncRNA H19 regulates *C1QTNF6*-mediated inflammation by sponging miR-29b in leukocytes, thereby promoting the activation and upregulation of IL-1β and TNF-α in these cells [14]. In breast cancer cells, CTRP6 expression is downregulated and exhibits a negative correlation with the levels of IL-6 and TNF-α. Treatment of MCF-7 cells with recombinant CTRP6 protein resulted in decreased cell viability and reduced expression of IL-6 and TNF-α [16]. Other studies have demonstrated that CTRP6 attenuates inflammation, oxidative stress, and apoptosis induced by cerebral ischemia/reperfusion through inhibition of the RhoA/Rock/PTEN pathway, thereby activating the PI3K/Akt signaling pathway [17]. In polycystic ovary syndrome (PCOS), *C1QTNF6* modulates cellular inflammatory responses via the AKT/NF-κB signaling pathway, thereby contributing to the pathogenesis of PCOS [18]. Inflammatory responses are a critical component of the immune response. During infection, these responses trigger the release of cytokines, which activate immune cells and initiate a coordinated immune defense. Concurrently, immune dysfunction may result in chronic inflammation or the development of autoimmune diseases. *C1QTNF6* modulates host immune tolerance and activation through regulation of immune cell functions, particularly in macrophages, dendritic cells, and T cells. Research indicates that *C1QTNF6* plays a critical role not only in antiviral immunity but also contributes to the pathogenesis of various immune-mediated diseases, including rheumatoid arthritis [12] and other autoimmune conditions [6]. Moreover, recent studies suggest that *C1QTNF6* exerts regulatory effects in antiviral defense [19], modulation of inflammatory responses [12,14,20], and maintenance of immune tolerance [21].

This study systematically characterized the tissue-specific expression profile of *C1QTNF6* in pigs and employed transcriptomic sequencing to analyze its regulatory network in porcine alveolar macrophages (3D4/21). Our findings demonstrate that *C1QTNF6* upregulation significantly induces antiviral-related gene expression in 3D4/21 cells. Using an established PCV2-infected 3D4/21 cell model, we further examined how *C1QTNF6* and its downstream target *MX2* influence viral infection. Notably, both *C1QTNF6* and *MX2* overexpression significantly suppressed PCV2 replication. Together, these results provide important insights into PCV2-host interactions and the development of novel antiviral strategies.

## 2. Materials and Methods

### 2.1. Cells and Experimental Reagents

Fetal bovine serum (FBS, BC-SE-FBS01C, Biochannel, Nanjing, China) and 1640 medium Gibco (12633020, Invitrogen Corporation, Carlsbad, CA, USA). MEM non-essential amino acids (11140035, Gibco, Carlsbad, CA, USA); L-glutamine (25030081, Gibco, Carlsbad, CA, USA); Bcl-2 antibody (15071s, CST 15071s, Shanghai, China); Bax antibody (89477S, CST); CAP antibody (GTX641316, Gentex, Sanantonio, TX, USA); *ISG15* antibody (Ab285367, Abcam, Cambridge, UK); Annexin V PE/7-AAD Apoptosis Detection Kit (CA1030-50T, Solarbio, CA1030-50T, Beijing, China); L-glutamine (HY-N0390, MCE, Shanghai, China).

### 2.2. The RNA of Different Tissue Samples

In previous work, our research group systematically collected samples from 12 pregnant commercial ternary (Duroc × Landrace × Yorkshire) sows, spanning four critical gestational stages (25d, 40d, 80d, and 112d). For each sow, we harvested multiple tissue types, including heart, liver, spleen, lung, kidney, leg muscle, duodenum, thymus, ovary, and placenta. Total RNA has been extracted from all collected tissues, while the remaining carcass portions were allocated to other research projects. The experiments were approved by the Animal Care and Use Committee of Yangzhou University (license approval number: SYXK (Su) 2021-0026).

### 2.3. Cell Culture, Virus Amplification

The 3D4/21 cells were cultured in 1640 medium supplemented with non-essential amino acids, L-glutamine (100 mg/mL) and 10% FBS, inoculated in tissue culture flasks, and placed in an incubator containing 5% CO_2_ at 37 °C. The PCV2d (MOI = 1) strain was preserved in our laboratory.

PK15 cells (ATCC, CCL-33) were cultured in Dulbecco’s Modified Eagle Medium (DMEM; Gibco, Waltham, MA, USA) supplemented with 10% fetal bovine serum (BC-SE-FBS01C, Biochannel, Nanjing, China) and 1% penicillin-streptomycin (100 µg/mL penicillin, 0.1 mg/mL streptomycin; Solarbio, Beijing, China).

### 2.4. Cell Transfection

The 3D4/21 cells from tissue culture flasks were digested when they were full grown and inoculated into 6-well plates at a density of 5 × 10^5^ cells/well, and the cells were transfected when the degree of fusion reached about 60%. In both groups, *C1QTNF6* overexpression and *MX2* overexpression plasmids were transfected, respectively, with pcDNA3.1 as the control, with the amount of 2000 ng/well. Add to 200 μL jet PRIME Buffer, shake and mix well, then add 3 μL jet PRIME (101000046/114-15, PolyPlus, Strasbourg, France), gently blow and mix well, and then incubate for 10min at room temperature. The transfection mixture was added to the wells, and 3 replicates were performed in each group.

### 2.5. Cellular DNA Extraction and RT-qPCR Analysis

Genomic DNA was extracted and purified using a commercial DNA extraction kit (FastPure Cell/Tissue DNA Isolation Mini Kit, DC102-01, Vazyme, Nanjing, China). The concentration and purity of the extracted DNA were measured with a Nanodrop ND-100 spectrophotometer, and the DNA was stored at −20 °C for subsequent analysis.

The viral copies analysis based on the calculation method reported in the previous literature [22,23,24]. The detailed operation steps are as follows: first, extract the total DNA of the cells and accurately determine its concentration. Then, 500 ng of total DNA was taken from each sample and diluted to the same volume (10 μL), and 1 μL of the diluted total DNA sample (50ng DNA) was taken for qPCR detection and analysis. We used a relative quantitative method to detect, comparing the viral DNA (Cap gene) with the host reference gene β-actin, and calculating the relative change factor (2^−ΔΔCt^).

### 2.6. Analysis of Apoptosis Using Flow Cytometry

The 3D4/21 cells were inoculated in 6-well plates and transfected (three replicates each of *C1QTNF6*-OE and pcDNA3.1) when the density hit 60%, placed in a cell culture box for 48 h and then subjected to apoptosis assay according to the scores of the kit instructions. Cells were digested with EDTA-free trypsin and collected into centrifuge tubes for spare parts. after centrifugation at 1000 rpm for 5 min, the supernatant was discarded and the cells were resuspended with 1 mL of pre-cooled PBS; after centrifugation at 1000 rpm for 5 min, the supernatant was discarded and the cells were resuspended by adding 500 μL of binding buffer, and 100 μL of the cell suspension was pipetted into new centrifuge tubes, and 5 μL of the cell suspension was added. The Annexin V-fluorescein isothiocyanate (FITC)/propidium iodide (PI) Apoptosis Detection Kit (CA1020, Solarbio, Beijing, China) was used to detect the cell apoptosis. Then, use flow cytometry to detect the cells according to the standard procedure, each measurement was performed three times using a total of 10,000 events per sample.

### 2.7. Total RNA Extraction and RT-PCR Analysis

Total RNA from 3D4/21 cells was extracted using Trizol reagent (9010, Takara, Dalian, China), and the concentration and purity were determined using ND-100 Nucleic Acid/Protein Concentration Meter. Total cellular RNA was used as a template for the synthesis of cDNA (RT101-01, Vazyme, Nanjing, China): 20 μL of the reaction system contained 4 μL of 4× HisyGO qRTRed Super mix, 3 μL of 5× gDNA Wiper Mix, 1000 ng of total RNA, and the RNase Free Water was added to 20 μL. The reaction procedure was 37 °C for 15 min, 85 °C for 5 s. The fluorescence quantification reaction system was 10 μL in an ABI Step OnePlus Real-Time PCR System (Applied Biosystems, Foster City, CA, USA), including 1 μL of cDNA, 0.1 μL of primers, 5 μL of 2× SuperStar Universal SYBR Master Mix (CW3360H, Cwbio, Beijing, China), and 3 μL of ddH_2_O. The program of the thermocycler was set as follows: reaction at 95 °C for 30 s; 95 °C for 15 s, 60 °C for 30 s, and 40 cycles. 60 °C for 30 s, and cycling for 40 times. Three independent experimental replicates were performed for all analyses. Primer information for the RT-qPCR in Table S4.

### 2.8. Western Blotting Analysis

Wash three times with pre-cooled PBS, add 200 μL of radioimmunoprecipitation assay (RIPA) (C1053, Applygen, Beijing, China) buffer containing with 1% protease inhibitor and phosphatase inhibitor were added to RIPA lysate, lysed for 20 min on ice, centrifuged at 12,000 r/min for 25 min at 4 °C, and aspirated the supernatant into a new 1.5 mL enzyme-free EP tube. The samples were homogenized by adding 5× SDS-PAGE (20315ES20, Yeasen Biotechnology, Shanghai, China) and heated at 98 °C for 10min followed by a rapid ice bath. Use a spiking needle to top up 20 μg of sample per well, run at constant pressure 70 V/25 min to the bottom of the concentrated gel, then run at 120 V until all of the bromophenol blue indicator is deposited on the bottom. Activated the PVDF (ISEQ0010, Millipore, Billerica, MA, USA) membrane by soaking it in methanol for 30 s. Use the ACE membrane transfer instrument to set the transfer time according to the protein size. The PVDF membrane was gently removed and labeled and placed in 5% skimmed milk powder (1× TBST preparation) and closed in a shaker at room temperature for 2 h. After completion of the closure, the membrane was cleaned with TBST buffer shaker for 3 times, each time for 10 min; the primary antibody of the target protein was added to PVDF membrane and closed in a shaker at 4 °C overnight. After the incubation was completed, the primary antibody was recovered and washed 3 times with 1× TBST shaker, each time for 10 min; the secondary antibody was added (the species was the same as the primary antibody) and incubated in shaker at room temperature for 2 h. After the incubation was completed, the secondary antibody was recovered and washed 3 times with 1× TBST, each time for 10 min. Preparation of Electrochemiluminescence (ECL) (New Cell & Molecular Biotech, Suzhou, China) Luminescence Developer Solution (ready to use, liquid A:liquid B = 1:1), gently remove the PVDF membrane with tweezers, place it on absorbent paper to absorb the TBST, then spread the ECL developing solution evenly on the membrane, incubate at room temperature and avoiding light for 5 min, and then develop the image by exposure with Tanon chemiluminescence imager; the experiment was conducted with the expression level of the HSP90 (60318–1-lg, Proteintech, Wuhan, China) or β-actin (ab8227, Abcam, Bridge, UK) proteins in the cells as the internal reference.

### 2.9. RNA-Seq Library Preparation and Data Analysis

Total six RNA samples were extracted using Trizol reagent (9010, Takara, Dalian, China), each group has 3 repetitions. Following fragmentation, mRNA was reverse-transcribed into cDNA and purified. cDNA fragments (~250–300 bp) were PCR-amplified and size-selected using the AMPure XP system (Beckman Coulter, Brea, CA, USA). Sequencing libraries were prepared and sequenced on an Illumina NovaSeq 6000 platform to generate 150 bp paired-end reads. Raw reads were quality-trimmed and adapter-removed using in-house Perl scripts, then aligned to the mouse reference genome (Release mm10) with STAR [25]. Gene counts were quantified with HTSeq [26], and expression levels (FPKM) were estimated by StringTie. Differential expression analysis was performed using DESeq2 [27], with genes satisfying |log2FC| ≥ 0.5 and adjusted *p* < 0.05 considered differentially expressed.

### 2.10. Data Analysis and Statistics

Fluorescence quantification results were normalized to the expression of the target genes using the housekeeping gene GAPDH, and relative quantification was presented as 2^−ΔΔCt^. Statistical analysis and graphing was performed using GraphPad 8.0.1. (GraphPad, San Diego, CA, USA) and data were presented as Mean ± SEM. Statistical analyses were performed by using unpaired two-tailed Student’s *t*-test or Multiple *t*-tests. * *p* < 0.05, ** *p* < 0.01, *** *p* < 0.001.

## 3. Results

### 3.1. Analysis of C1QTNF6 Expression Profiles Across Porcine and Human Tissues

**Figure 1:**
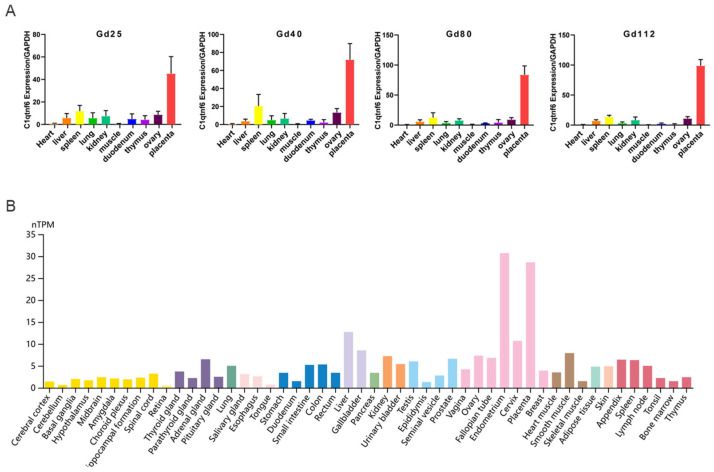
*C1QTNF6* expression characteristics. (**A**) *C1QTNF6* expression across various pig tissues at different stages of gestation. *n* = 3. Gd25: Gestation day 25, Gd40: Gestation Day 40, Gd80: Gestation Day 80, Gd112: Gestation Day 112. (**B**) Analysis of *C1QTNF6* protein expression profile based on protein profiles of human tissues.

Initially, we first characterized the tissue expression profile of *C1QTNF6* in pigs and observed its specific and high expression in placental tissue. Compared to heart tissue, *C1QTNF6* expression was significantly upregulated in spleen, lung, and kidney tissues (Figure 1A). Consistent with this, analysis of the Human Protein Atlas also revealed specific enrichment of *C1QTNF6* in human placental tissue, indicating a conserved expression pattern across species (Figure 1B). Notably, elevated *C1QTNF6* expression was also detected in other human immune-related tissues—including liver, lung, thymus, and bone marrow—suggesting a potential role for *C1QTNF6* in immune regulation in pigs.

### 3.2. C1QTNF6 Significantly Modulates the Proliferative Activity of 3D4/21 Cells

**Figure 2:**
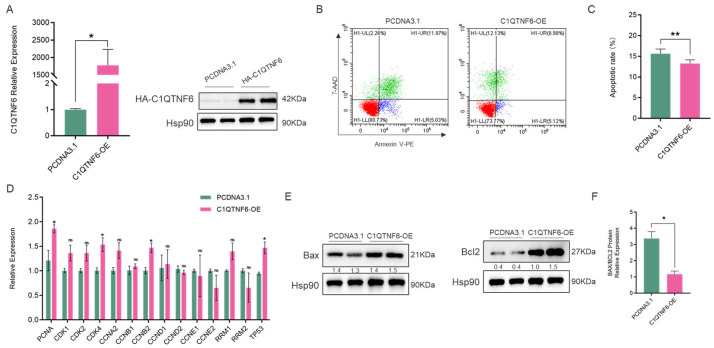
*C1QTNF6* significantly modulates the proliferative activity of 3D4/21 cells. (**A**) RT-PCR (*n* = 3) and Western blotting detected the efficiency of *C1QTNF6* overexpression, *n* = 2. (**B**,**C**) Flow cytometry analysis to detect apoptosis in 3D4/21 cells after *C1QTNF6* overexpression, *n* = 3. (**D**) RT-PCR to detect expression changes in cycle-related genes after *C1QTNF6* overexpression. (**E**,**F**) Western blotting analysis the expression of apoptosis-related protein (Bax and Bcl2), *n* = 2. Statistical analyses were performed by using unpaired two-tailed Student’s *t*-test. ns: No significantly. * *p* < 0.05, ** *p* < 0.01.

The porcine alveolar macrophages cell line (3D4/21) was used as a cellular model to investigate the potential function of *C1QTNF6*. We first constructed a *C1QTNF6* overexpression vector and confirmed its high transfection efficiency (Figure 2A). Flow cytometry analysis revealed that the overexpression of *C1QTNF6* significantly reduced apoptosis in 3D4/21 cells (Figure 2B,C). RT-qPCR analysis revealed that overexpression of *C1QTNF6* significantly upregulated the mRNA levels of *PCNA*, *CDK4*, *CCNB2* and *TP53* key cycling factors (Figure 2D), and also up-regulated the expression of anti-apoptotic protein BCL2 (Figure 2E), the ratio of Bax/BCL2 was decreased (*p* < 0.05) (Figure 2F). These results indicated that the overexpression of *C1QTNF6* might enhance the anti-apoptotic effect of 3D4/21 cells.

### 3.3. C1QTNF6 Significantly Upregulates the Expression of Pro-Inflammatory Immune Genes

**Figure 3:**
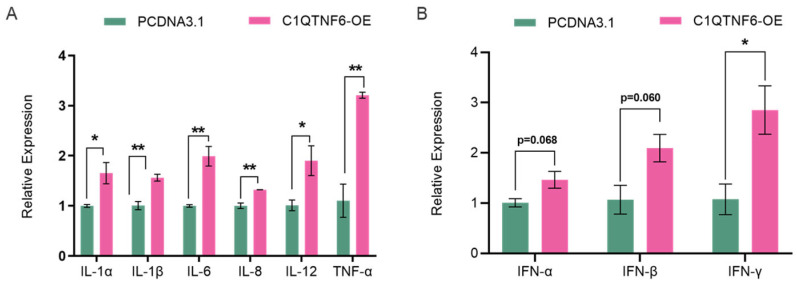
*C1QTNF6* significantly promotes the expression of immune-related genes. (**A**) RT-PCR to detect changes in inflammatory factor-related gene expression after *C1QTNF6* overexpression. *n* = 3. (**B**) RT-PCR to detect changes in interferon-related gene expression after *C1QTNF6* overexpression, *n* = 3. Statistical analyses were performed by using unpaired two-tailed Student’s *t*-test. * *p* < 0.05, ** *p* < 0.01.

We examined the changes in mRNA expression levels of cytokines in cells after overexpression of *C1QTNF6* and found that the levels of pro-inflammatory factors *IL-1α* and *IL-1β* were elevated (Figure 3A). These two pro-inflammatory factors are produced by interleukin-1 (IL-1), which binds to specific IL-1 receptors and activates the NF-κB and MAPK signaling pathways through MyD88, IRAK4, and TRAF6, subsequently promoting the transcription of various inflammatory genes [28,29]. Elevated expressions of *IL-6*, *IL-8*, *IL-12* and *TNF-α* were involved in the cell immune responses (Figure 3A). Interferons and the interleukin family members are cytokines produced by immune cells, serving as endogenous signals for cell-to-cell interactions. These cytokines can also be secondary to the induction of other cytokines, facilitating interactions among immune cells [30]. Due to the varying degrees of elevation within the interleukin family, we assessed the expression levels of interferons and observed increased levels of *IFN-α*, *IFN-β* and a notably elevated level of *IFN-γ* (Figure 3B).

### 3.4. C1QTNF6 Overexpression Upregulates Immune-Related Gene Expression in 3D4/21 Cells

**Figure 4:**
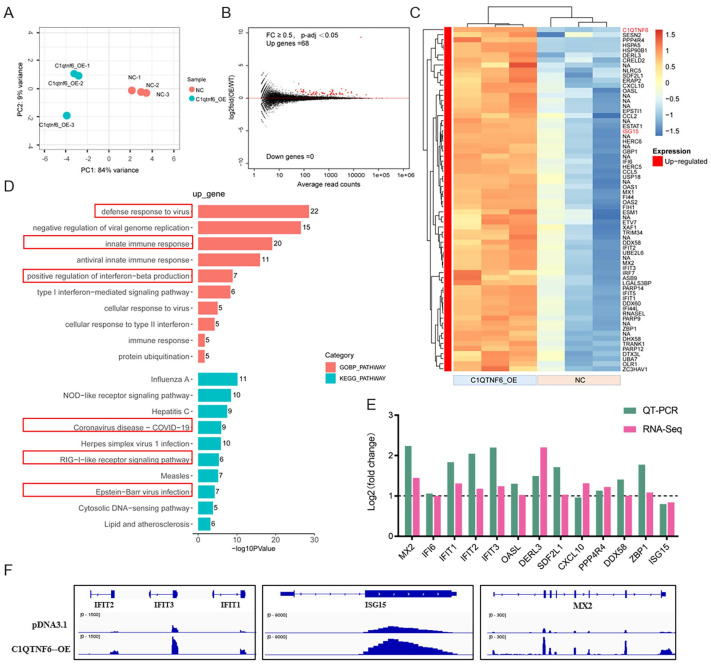
*C1QTNF6* overexpression upregulates immune-related gene expression in 3D4/21 cells. (**A**) PCA group clustering analysis after *C1QTNF6* overexpression. (**B**) Gene volcano map after *C1QTNF6* overexpression. (**C**) Heat map of 68 genes after *C1QTNF6* overexpression. FC ≥ 0.5 and *p*-adj < 0.05. (**D**) Functional enrichment analysis results of up-regulated expressed genes after *C1QTNF6* overexpression, only Top10 entries are shown. (**E**) RT-PCR validation of differentially expressed genes screened by RNA-Seq, *n* = 3. (**F**) IGV showing representative genes with altered mRNA expression levels after *C1QTNF6* overexpression.

To further explore the role of *C1QTNF6*, an analysis of the transcriptome data following its overexpression was conducted (Figure 4A,B). Interestingly, standard analysis revealed a total of 68 differentially expressed genes, all of which were upregulated. (Figure 4C, Table S1). GO functional enrichment analysis indicated that the up-regulated genes were predominantly enriched in pathways associated with antiviral responses, innate immune responses, COVID-19 disease mechanism, and the RIG-I pathway (Figure 4D, Tables S2 and S3), all of which are fundamentally related to antiviral activities. We conducted RT-PCR validation on 13 of these genes, and the results were consistent with those obtained from RNA-Seq analysis (Figure 4E). IGV demonstrated the changes in genes that might be regulated by *C1QTNF6* (Figure 4F). These findings collectively suggest that *C1QTNF6* may play a critical role in host defense against viral infection.

### 3.5. PCV2 Infection Markedly Downregulates C1QTNF6 Expression in 3D4/21 Cells

**Figure 5:**
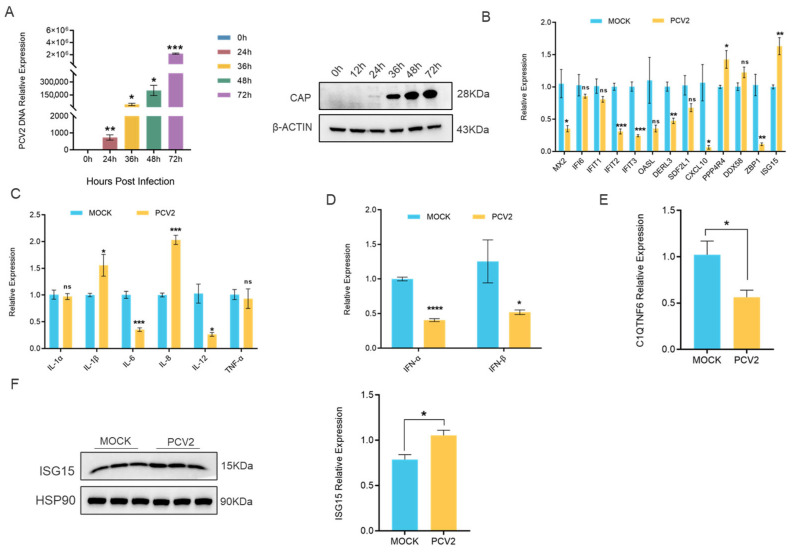
PCV2 infection markedly downregulates *C1QTNF6* expression in 3D4/21 cells. (**A**) Expression analysis of CAP genes (*n* = 3) and protein at different time points in PCV2-infected 3D4/21 cells. Statistical analyses were performed by multiple *t*-test. (**B**) Alterations in *C1qtnf6*-regulated gene expression following 48 h PCV2 infection in 3D4/21 cells, *n* = 3. (**C**) Alterations in inflammatory factor-related genes expression following 48 h PCV2 infection in 3D4/21 cells, *n* = 3. (**D**) Alterations in interferon-related genes expression following 48 h PCV2 infection in 3D4/21 cells, *n* = 3. (**E**) Expression changes in *C1qtnf6* following 48 h PCV2 infection in 3D4/21 cells, *n* = 3. (**F**) Changes in the expression of *ISG15* protein after PCV2 infection of cells for 48h, *n* = 3. Statistical analyses were performed by using unpaired two-tailed Student’s *t*-test. ns: No significantly. * *p* < 0.05, ** *p* < 0.01, *** *p* < 0.001, **** *p* < 0.0001.

To investigate the role of *C1QTNF6* in viral infection, we established an in vitro model of PCV2 infection by treating 3D4/21 cells with 1 multiplicity of infection (MOI) of PCV2 and confirmed the infection by staining of the viral CAP protein (Figure 5A). Firstly, we investigated the expression levels of 13 genes that are significantly regulated by *C1QTNF6* following PCV2 infection, and we found that *MX2*, *IFIT2*, *IFIT3*, *CXCL10* and *ZBP1* were notably downregulated, while *ISG15* exhibited a significant increase (Figure 5B). On this basis, we investigated the expression levels of genes associated with inflammatory factors and interferon. The results indicated that PCV2 infection led to decrease in mRNA levels of *IL-6*, *IL-12*, *IFN-α* and *IFN-β*, while simultaneously increasing mRNA levels of *IL-8* (Figure 5C,D). These findings indicate that PCV2 infection induces immunosuppression in the host and selectively upregulates *IL-8* mRNA levels, demonstrating that it can cause immune dysfunction and trigger an inflammatory response. It was noted that the differentially expressed gene was primarily enriched in the antiviral pathway following the overexpression of *C1QTNF6*. The results from RT-PCR indicated that the mRNA level of *C1QTNF6* decreased subsequent to PCV2 infection. (Figure 5E). The expression of interferon-stimulated factor *ISG15* was elevated (Figure 5F). These results suggest that *C1QTNF6* may be involved in the host immune response due to PCV2 infection.

### 3.6. C1QTNF6 Inhibits PCV2 Replication in Association with MX2 Upregulation

**Figure 6:**
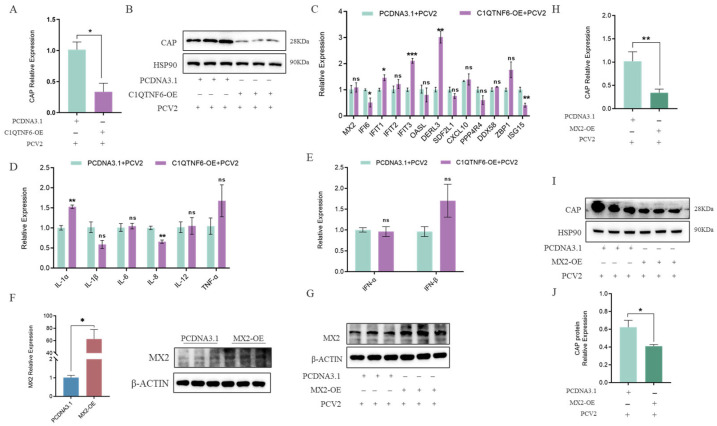
*C1QTNF6* inhibited PCV2 virus replication by upregulating the *MX2* gene. (**A**,**B**) Expression of PCV2 Cap gene and protein in *C1QTNF6* overexpressing 3D4/21 cells after PCV2 infection, *n* = 3. (**C**) Differential expression of *C1QTNF6* regulated genes at 48 h post infection in *C1QTNF6* overexpressing cells, *n* = 3. (**D**) Inflammatory cytokine expression in PCV2 infected 3D4/21 cells overexpressing *C1QTNF6*, *n* = 3. (**E**) Interferon related gene expression in PCV2 infected 3D4/21 cells overexpressing *C1QTNF6*, *n* = 3. (**F**) Validation of *MX2* overexpression efficiency by RT qPCR and Western blotting, *n* = 3. (**G**) *MX2* expression in *MX2* overexpressing 3D4/21 cells at after PCV2 infection, *n* = 3. (**H**–**J**) PCV2 Cap mRNA and protein expression in *MX2*-overexpressing 3D4/21 cells at 48 h post-infection, *n* = 3. Statistical analyses were performed by using unpaired two-tailed Student’s *t*-test. ns: No significantly. * *p* < 0.05, ** *p* < 0.01, *** *p* < 0.001.

To further investigate the potential antiviral effect of *C1QTNF6*, 3D4/21 cells were infected with PCV2 following *C1QTNF6* overexpression. The results revealed a significant reduction in both PCV2 DNA copy number and Cap protein expression levels (Figure 6A,B). In contrast to PCV2 infection alone, after *C1QTNF6* overexpression led to elevated expression of interferon-stimulated genes (ISGs) including *MX2*, *IFIT3*, *ZBP1*, and *DERL3*, while *ISG15* and *CXCL10* were downregulated (Figure 6C), which might be closely related to the ability to inhibit the replication of PCV2. Expression of interleukin-related and interferon-related genes was also markedly altered: *IL-6* mRNA levels increased, whereas *IL-8* levels decreased (Figure 6D), and the PCV2-induced suppression of *IFN-α* and *IFN-β* was reversed (Figure 6E). These findings suggest that *C1QTNF6* may restore the host inflammatory and antiviral responses in porcine macrophages during PCV2 infection by activating interferon-stimulated pathways. Given that *MX2* is a canonical interferon-stimulated antiviral factor among porcine ISGs [31], we hypothesize that the antiviral effect of *C1QTNF6* may be related to the upregulation of *MX2*. To test this, an *MX2* overexpression vector was constructed and validated in 3D4/21 cells by RT-qPCR (Figure 6F,G). Subsequent PCV2 infection in *MX2*-overexpressing cells resulted in significantly reduced viral DNA copy number and Cap protein levels compared to PCV2 infection alone (Figure 6H–J), confirming the antiviral role of *MX2*. Collectively, these results demonstrate that *C1QTNF6* inhibits PCV2 replication in 3D4/21 cells, at least in part through the upregulation of *MX2*, thereby highlighting a novel mechanism by which *C1QTNF6* contributes to innate antiviral immunity.

## 4. Discussion

The C1QTNF (C1q/TNF-related protein) family belongs to the C1q/TNF-related protein superfamily, commonly referred to as the CTRP superfamily. Members of this family share structural domains homologous to those of complement component C1q and tumor necrosis factor (TNF) [4], implicated in both immune [31] and metabolic regulatory functions [6]. To date, at least 15 members have been identified in humans and other mammals, designated C1QTNF1–C1QTNF15 and correspondingly termed CTRP1–CTRP15 [5]. C1QTNF proteins typically consist of four distinct domains: an N-terminal signal peptide, a variable region, a collagen-like domain, and a C-terminal globular C1q domain [32]. The globular C1q domain exhibits structural similarity to domains found in C1q and TNF family proteins [33], mediates receptor binding and signal transduction, and functions as a secreted cytokine in vivo. *C1QTNF6* is predominantly expressed in human synovial cells, placenta, and submandibular glands, with the highest expression levels observed in the placenta [12,34]. This expression pattern is consistent with the tissue distribution of *C1QTNF6* observed in pigs in the present study. Nevertheless, the functional roles of *C1QTNF6* in porcine cells remain poorly characterized.

PCV2 is a prototypical member of the genus Circovirus within the family Circoviridae and represents the primary etiological agent of porcine circovirus-associated diseases (PCVAD) [35]. PCV2 targets and damages key immune organs, including lymph nodes, spleen, and tonsils [31,36]—thereby impairing host immune function. This immunosuppression predisposes infected animals to secondary infections with bacteria, viruses, or mycoplasmas, which often exacerbate clinical outcomes. Consequently, PCV2 infection leads to reduced growth performance, increased culling rates, and compromised vaccine efficacy, resulting in substantial direct and indirect economic losses in the swine industry, particularly during high-density rearing, weaning, or transition phases. Owing to its high pathogenicity, widespread prevalence, and significant economic impact, PCV2 has become a major focus of veterinary research and disease control efforts [37]. Elucidating the molecular mechanisms underlying host resistance to PCV2 infection—particularly the roles of innate immune regulators such as *C1QTNF6*—is therefore of considerable significance for the development of novel intervention strategies and the improvement of disease management. The present study aimed to investigate the functional role of *C1QTNF6* in porcine alveolar macrophages (3D4/21 cells) during PCV2 infection. Initial analyses revealed that *C1QTNF6* expression significantly suppressed apoptosis and enhanced cellular proliferative activity, accompanied by marked upregulation of proliferation-associated genes. However, the molecular basis for these effects remained unclear.

To address this, transcriptomic profiling was performed following *C1QTNF6* overexpression. Only 68 genes were significantly upregulated, the majority of which were identified as interferon-stimulated genes (ISGs). This finding underscores a potential role for *C1QTNF6* in immune regulation within porcine cells. Consistent with prior reports, *C1QTNF6* has been shown to activate innate immune cells such as macrophages and monocytes, modulate the secretion of proinflammatory cytokines—including TNF-α, IL-6, and IL-1β—and potentially engage downstream signaling pathways such as JAK/STAT, NF-κB, or IRF to induce antiviral gene expression [38]. Gene enrichment analysis further revealed that *C1QTNF6*-upregulated genes were significantly enriched in biological processes related to defense response to viruses, negative regulation of viral genome replication, innate immune response, and antiviral innate immune response, supporting the notion that *C1QTNF6* activates interferon-related pathways in 3D4/21 cells and may confer antiviral activity.

To validate this hypothesis, a PCV2-infected 3D4/21 cell model was established. Infection with PCV2 led to a marked reduction in *C1QTNF6* mRNA levels, concomitant with significant downregulation of key ISGs, including *MX2*, *IFIT3*, *CXCL10* and *ZBP1*. These results corroborate the well-documented immunosuppressive nature of PCV2 [39,40]. Previous studies have indicated that PCV2 pathogenesis involves complex virus–host interactions that exacerbate disease through immune suppression and dysregulated inflammatory responses [41].

PCV2 utilizes host surface receptors such as CD163 to gain entry into target cells, primarily infecting lymphoid tissues and the lungs [39,40]. The virus replicates within macrophages and dendritic cells via a rolling-circle mechanism [42,43]. Viral replication is governed by the Rep protein, while the Cap protein mediates capsid assembly, ultimately contributing to cellular dysfunction. Notably, PCV2 exhibits selective immunosuppressive effects by inducing apoptosis in B cells and CD4^+^ T cells. Experimental data demonstrate that PCV2 infection can reduce peripheral blood CD4^+^ T cell counts by up to 50% and B cell counts by approximately 30% [39,40], thereby establishing a state of systemic immunosuppression.

In contrast, *C1QTNF6* overexpression restored the expression of ISGs—including *MX2*, *IFIT2*, *IFIT3*, *CXCL10* and *ZBP1*—and significantly inhibited PCV2 replication. These findings further support the antiviral capacity of *C1QTNF6*. Comparative analysis of differentially expressed genes revealed that *C1QTNF6* expression effectively mitigated the PCV2-induced downregulation of *MX2*, a canonical interferon-stimulated gene. This observation suggests that *C1QTNF6* may exert its antiviral effects, at least in part, through the induction of *MX2*. Among the 14 genes exhibiting highly significant differential expression following *C1QTNF6* overexpression, several are well-established interferon-induced effectors, including *IFIT3*, *MX2*, *ISG15* and *OASL* [38]. *MX2*, a member of the myxovirus resistance (MX) protein family, plays a critical role in cellular antiviral immunity and is a key effector of the type I interferon (IFN-I) signaling pathway [44,45,46]. In the present study, *MX2* expression was upregulated upon *C1QTNF6* overexpression but suppressed following PCV2 infection. Prior studies have demonstrated that *MX2* interferes with multiple stages of the viral life cycle, thereby inhibiting replication in host cells [47]. *MX2* expression is primarily induced by IFN-α and IFN-β [48], and interferon-induced *MX2* has been shown to restrict the replication of porcine reproductive and respiratory syndrome virus (PRRSV) [48]. Activation of the interferon signaling cascade leads to *MX2* upregulation, enhancing antiviral defense. The central function of *MX2* is to reduce viral load by inhibiting viral entry or replication [49,50]. For instance, during HIV infection, *MX2* binds to the viral capsid core to block reverse transcription [51]; in PRRSV, *MX2* interacts with the viral nucleocapsid (N) protein. Notably, the N-terminal 51 amino acids of porcine *MX2* appear dispensable for its anti-PRRSV activity, in contrast to human *MX2*, where residues 1–29 are essential [50]. It is well established that apoptosis serves as a host defense mechanism that effectively interrupts viral replication and eliminates infected cells [52]. Our study found that *C1QTNF6* overexpression significantly inhibits apoptosis and influences cell cycle-related factors. This raises a critical question: could enhanced cellular activity actually promote viral replication? research indicates that PCV2 employs a dual strategy to evade apoptosis: on one hand, it upregulates anti-apoptotic factors (e.g., SERPINB9) to delay the death of infected cells, thereby gaining time for viral replication [52]; on the other hand, it activates the PERK–ROS–p53 pathway to induce S phase cell cycle arrest, exploiting the enriched pool of DNA replication substrates and enzymes during this phase to efficiently synthesize its viral genome [53]. However, our study revealed that *C1QTNF6* overexpression instead significantly suppressed PCV2 replication. This seemingly paradoxical finding constitutes a central scientific question of our study and points to a key direction for future research: to elucidate the precise mechanism by which *C1QTNF6* effectively inhibits PCV2 replication while concurrently exerting its own anti-apoptotic activity.

**Figure 7:**
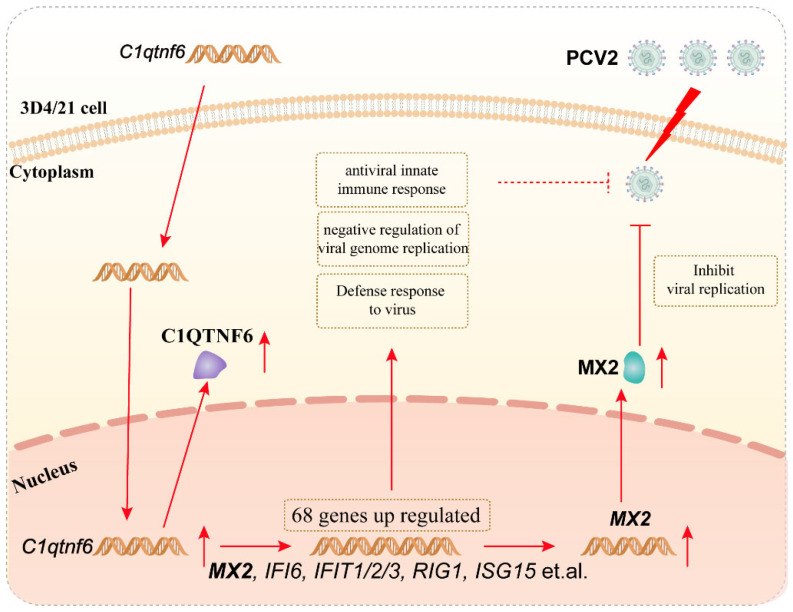
Mechanism of *C1QTNF6* inhibiting PCV2 replication in association with *MX2* upregulation.

Based on these observations, it is hypothesized that the antiviral activity of *C1QTNF6* against PCV2 is mediated, at least partially, through the upregulation of *MX2*. To test this, *MX2* was overexpressed in 3D4/21 cells, resulting in a significant reduction in both Cap gene transcription and Cap protein expression levels of PCV2. This confirms that *MX2* exerts a direct antiviral effect against PCV2 infection. Collectively, these findings support the conclusion that *C1QTNF6* inhibits PCV2 replication, at least in part, by inducing the expression of *MX2* (Figure 7).

## 5. Limitations of the Study

Several limitations of this study should be noted. First, although *C1QTNF6* overexpression significantly upregulates genes in antiviral pathways—with *MX2* expression aligning with the antiviral effects observed—it remains unclear whether *MX2* is the primary effector responsible for *C1QTNF6*’s function. Performing knockdown or knockout experiments targeting *MX2* under conditions of *C1QTNF6* overexpression could help establish a direct functional link between *C1QTNF6* and *MX2*. Second, the mechanism by which overexpression of *C1QTNF6* inhibits PCV2 replication in the context of inhibiting apoptosis remains unclear. Further studies based on cell and animal models involving apoptosis-related proteins can provide more evidence for the antiviral contribution of *C1QTNF6*. Last, given that the current data are entirely in vitro in 3D4/21 cell line, further validation in primary porcine alveolar macrophages or ex vivo lung-derived macrophages would more effectively substantiate these findings and better reflect their relevance for in vivo PCV2-associated diseases and the development of livestock vaccines or antivirals. Future research will focus on these limitations to further verify the antiviral function and mechanism of *C1QTNF6* as a host factor.

## Data Availability

The raw data supporting the conclusions of this article will be made available by the authors on request.

## Supplementary Materials

The following supporting information can be downloaded at: https://www.mdpi.com/article/10.3390/vetsci13010011/s1, Table S1: Differentially expressed genes between treatment and negative control groups, Table S2: List of the upregulated expressed gene; Table S3: List of KEGG pathways obtained by analysis; Table S4: Primer information for the RT-qPCR.

## Abbreviations

The following abbreviations are used in this manuscript:

*C1QTNF6*	C1q and tumor necrosis factor-related protein 6
PCV2	Porcine Circovirus Type 2
ISGs	Interferon-stimulated genes
3D4/21	Porcine alveolar macrophages cell line
PCVAD	Porcine Circovirus-Associated Diseases
PRRSV	Porcine reproductive and respiratory syndrome virus
