# Supplementary material for: The C1QTNF6–MX2 Antiviral Axis Inhibits Porcine Circovirus Type 2 Replication in Porcine Macrophages
Source: Vet Sci. 2025 Dec 21;13(1):11. doi: 10.3390/vetsci13010011 (PMC12846478; doi:10.3390/vetsci13010011)
Supplement: Supplementary file 1 [file vetsci-13-00011-s001.zip › Table S4 Primes.pdf]

Table S4 Cell cycle-related genes

Primes	Forward (5' to 3')	Reverse (5' to 3')
PCNA	TGCAGATGTACCCCTTGTTGT	CATCTTCGATCTTGGGAGCCA
CDK1	AGGTCAAGTGGTAGCCATGAA	TCCATGAACTGACCAGGAGG
CDK2	AAAAGATCGGAGAGGGCACG	GCAGTACTGGGTACACCCTC
CDK4	CCTCCCGGTATGAACCAGTG	TGCTCAAACACCAGGGTCAC
CCNA2	GTACTGAAGGCCGGGAACTC	AGCTGGCCTCTTTTGAGTCT
CCNB1	ACGGCTGTTAGCTAGTGGTG	GAGCAGTTCTTGGCCTCAGT
CCNB2	TGGAAATCGAGTTACAACCAGA	TGGAGCCAACATTTCCATCTGT
CCND1	CTTCCATGCGGAAGATCGTG	TGGAGTTGTCGGTGTAGATGC
CCND2	TTCCCCAGTGCTCCTACTTC	CACAACTTCTCAGCCGTCAG
CCNE1	AGCCTGTGAAAACCCCTGTT	TCCAGAAGAATCGCTCGCAT
CCNE2	GGGGGATCAGTCCTTGCATT	AGCCAAACATCCTGTGAGCA
RRM1	CTTCAATGCTGGCACCAACC	TGTTTCCACCCTGATCCACG
RRM2	CATCGAGACAATGCCTTGCG	AGGCGAAGTCGCAGTGTAAA
TP53	ACGCTTCGAGATGTTCCGAG	TTTTATGGCGGGAGGGAGAC

Table S4 Cell cycle-related genes

Primes	Forward (5' to 3')	Reverse (5' to 3')
**IL-α**	**ACCTGGATGAGGCAGTGAAAT**	**ATGGGCGGCTGATTTGAAGT**
**IL-1β**	**AGGGACATGGAGAAGCGATTT**	**TTCTGCTTGAGAGGTGCTGATG**
**IL-6**	**CCTCGGCAAAATCTCTGCAA**	**TGAAACTCCACAAGACCGGT**
**IL-8**	**CCACACCTTTCCACCCCAAA**	**TTGTTGCTTCTCAGTTCTCTTCA**
**IL-12**	**CAGGCCCAGGAATGTTCAAA**	**CGTGGCTAGTTCAAGTGGTAAG**
**IFN-α**	**TTCTGCACTGGACTGGATC**	**TCTGTGGAAGTATTTCCTCACAG**
**IFN-β**	**GCTAACAAGTGCATCCTCCAAA**	**CCAGGAGCTTCTGACATGCCA**
**TNF-α**	**CGACTCAGTGCCGAGATCAA**	**CTCACAGGGCAATGATCCCA**

Table S4 Cell cycle-related genes

Primes	Forward (5' to 3')	Reverse (5' to 3')
**MX2**	**CCAGAGGCAGCGGAATCAT**	**TTTGCGTATTTCCCGCTCCA**
**IFI6**	**CGGTGGAGAGGAGACAGACA**	**TCGAGTTGCTTGCTGACAGT**
**IFIT1**	**GGTCTTGGAGGAGATTGAG**	**TAACCAGCCTTCTCACCTC**
**IFIT3**	**CCGCCATCATGAGTGAGGTC**	**TCATGCCAGACATGTTCTTCCT**
**OASL**	**CACCTTCGTGGCTAAATGCC**	**GAAGGAGCCCACCTCTAGC**
**DERL3**	**GCTCTACTTCAACCCGCACC**	**CAGTAGCGGAACACGAAGAG**
**SDF2L1**	**GTCCAACAACCAGGAGGTGAG**	**ACGGATGGGACTCCCGTATT**
**CXCL10**	**ACTGTTCGCTGTACCTGCAT**	**TCGAGGAGATCTTTTAGACCTTTCT**
**PPP4R4**	**GTTTATCTGCTCAGTGCTGGTC**	**CTGCCTTCAACATCTGGGACT**
**DDX58**	**GGAGATGCTTTCAGGGAGCG**	**GCAGTCTGGCCTAGCACAATA**
**ZBP1**	**GAAATCAGTCCACCCCGGTT**	**AGCCATGGAGCCGCTTTC**
**ISG15**	**CAGAGACCCACTGAGCATCC**	**GCGTCAGCCAGACCTCATAG**

**CAP**		
**β-Actin**		
**C1QTNF6**	**GTCTGCCCTGCCCTATGTG**	**CGGTCCCCTTTGCCCTTTAG**
